# Reliability of brain atrophy measurements in multiple sclerosis using MRI: an assessment of six freely available software packages for cross-sectional analyses

**DOI:** 10.1007/s00234-023-03189-8

**Published:** 2023-08-01

**Authors:** David R. van Nederpelt, Houshang Amiri, Iman Brouwer, Samantha Noteboom, Lidwine B. Mokkink, Frederik Barkhof, Hugo Vrenken, Joost P. A. Kuijer

**Affiliations:** 1 MS Center Amsterdam, Radiology and Nuclear Medicine, Vrije Universiteit Amsterdam, Amsterdam Neuroscience, Amsterdam UMC Location VUmc, Amsterdam, The Netherlands; 2 Neuroscience Research Center, Institute of Neuropharmacology, Kerman University of Medical Sciences, Kerman, Iran; 3 MS Center Amsterdam, Anatomy and Neurosciences, Vrije Universiteit Amsterdam, Amsterdam Neuroscience, Amsterdam UMC Location VUmc, Amsterdam, The Netherlands; 4 Department of Epidemiology and Data Science, Amsterdam Public Health Research Institute, Amsterdam UMC, Vrije Universiteit Amsterdam, 1007MB Amsterdam, The Netherlands; 5 Institutes of Neurology and Healthcare Engineering, UCL London, London, UK

**Keywords:** Brain volumetry, Multiple sclerosis, Segmentation, Reliability

## Abstract

**Purpose:**

Volume measurement using MRI is important to assess brain atrophy in multiple sclerosis (MS). However, differences between scanners, acquisition protocols, and analysis software introduce unwanted variability of volumes. To quantify theses effects, we compared within-scanner repeatability and between-scanner reproducibility of three different MR scanners for six brain segmentation methods.

**Methods:**

Twenty-one people with MS underwent scanning and rescanning on three 3 T MR scanners (GE MR750, Philips Ingenuity, Toshiba Vantage Titan) to obtain 3D T1-weighted images. FreeSurfer, FSL, SAMSEG, FastSurfer, CAT-12, and SynthSeg were used to quantify brain, white matter and (deep) gray matter volumes both from lesion-filled and non-lesion-filled 3D T1-weighted images. We used intra-class correlation coefficient (ICC) to quantify agreement; repeated-measures ANOVA to analyze systematic differences; and variance component analysis to quantify the standard error of measurement (SEM) and smallest detectable change (SDC).

**Results:**

For all six software, both between-scanner agreement (ICCs ranging 0.4–1) and within-scanner agreement (ICC range: 0.6–1) were typically good, and good to excellent (ICC > 0.7) for large structures. No clear differences were found between filled and non-filled images. However, gray and white matter volumes did differ systematically between scanners for all software (*p* < 0.05). Variance component analysis yielded within-scanner SDC ranging from 1.02% (SAMSEG, whole-brain) to 14.55% (FreeSurfer, CSF); and between-scanner SDC ranging from 4.83% (SynthSeg, thalamus) to 29.25% (CAT12, thalamus).

**Conclusion:**

Volume measurements of brain, GM and WM showed high repeatability, and high reproducibility despite substantial differences between scanners. Smallest detectable change was high, especially between different scanners, which hampers the clinical implementation of atrophy measurements.

**Supplementary Information:**

The online version contains supplementary material available at 10.1007/s00234-023-03189-8.

## Introduction

Multiple sclerosis (MS) is an autoimmune disease of the central nervous system that is characterized by demyelination, visible as focal lesion, and neurodegeneration, observable as atrophy of the spinal cord and brain, which is present from the earliest stages and more prominently in the progressive stages of the disease [1]. The inflammation component in MS is generally well suppressed with disease-modifying therapies; however, brain volume change, as a proxy of neurodegeneration, has gained increased attention to further facilitate treatment (response) monitoring and prognosis of the individual patient [2, 3]. In addition, atrophy is strongly linked to clinical and cognitive disability [4–6]. Brain atrophy in people with MS occurs at a faster rate (approximately 0.5–1.35% per year) than in healthy aging subjects [7, 8]. While early treatments in MS had limited effect on brain atrophy, recently developed MS treatments showed reduced brain atrophy rates [9, 10]. Moreover, a recent study [11] indicated that brain atrophy is associated with disease progression which was independent of relapse activity. This highlights the importance of developing reliable atrophy measurements in the clinic. Brain atrophy measurement using magnetic resonance imaging (MRI) is a way to assess disease progression and monitor treatment response in MS [8, 12].

Automated brain segmentation techniques have enabled efficient and reproducible processing of MR images. However, brain volumetry in MS is still challenging, e.g. due to differences in MR scanners, acquisition protocols, and analysis software. Differences between scanners include technological differences between vendors, models and field strength [13]. These effects are more pronounced in multi-center trials and especially in the clinical setting, where MR scanners and acquisition protocols can vary frequently, compared to single-center trials where sources of variation are better standardized.

Choice of acquisition method, including scanner model, and of analysis software affect the resulting volume measurements, as shown for limited number of scanners or vendors (usually 2) and mostly limited numbers of analysis software (1–4, but up to 7 for Durand-Dubief) [14–18]. Furthermore, most brain volume reproducibility studies have generally been performed for other disease types (such as Alzheimer’s Disease) or in healthy controls [19–21]. Additionally, the effect on detection of group differences is unclear. There are multiple freely available software packages for volume measurements on MR images [22–25]. Deep learning approaches have recently gained an increased interest in the field of brain volumetry and have shown promising results compared to traditional methods [26, 27]. New methods have generally been developed to be more robust for image contrast changes, however, within and between-scanner effects have not been studied, yet [28, 29]. Quantifying the effect of scanner used on the output of each software will provide an improved understanding of the resulting variability in atrophy measures, as well as mitigation of that variability. Moreover, it would be useful for future patient studies, to have an indication of the minimum real volume change that can be detected within one subject on one, or multiple, scanner(s). Similarly, when multiple scanners are used, an indication for the between-group differences and power could be beneficial.

In the current study, we applied six freely available brain volume segmentation techniques, including two novel techniques designed to be robust for image contrast, on whole-brain 3D T1-weighted (T1w) scans of 21 people with MS acquired using MR scanners from three different vendors, to examine the impact on both model-based and supervised machine learning algorithms. All subjects were scanned twice on the same scanner to assess the within-scanner repeatability. Besides within-scanner repeatability, we aim to quantify the between-scanner reproducibility of the volume measurements through evaluation of agreement between the MR scanners. Additionally, we evaluate the effect of lesion filling on repeatability and reproducibility.

## Materials and methods

### Participants

Baseline scans from a 1-year follow-up study in 21 subjects with MS (relapsing remitting MS *n* = 16; secondary progressive MS *n* = 1; and primary progressive MS *n* = 4), diagnosed according to McDonald 2010 criteria [30] were obtained between November 2016 and February 2017. Subjects included were between 18 and 70 years old. Exclusion criteria were any neurological/neuropsychological comorbidity and contraindication to undergo MRI examination. The subjects underwent a scan and rescan, hereafter referred to as first (scan) and second (rescan) run, on three 3 T MR scanners in the same center. Different MR examinations were performed on the same day or with a maximum of eight days between the scans. Between the first and second run the subjects got of the scanner bed and walked a few steps before repositioning. The institutional review board approved the study protocol (NL555598.029.15) and written informed consent was obtained from all individuals, according to the Declaration of Helsinki.

### MRI protocol

Patients were scanned on the following scanner: 1) 3 T GE Discovery MR750 (GE Healthcare, Milwaukee, USA), 2) 3 T Philips Ingenuity (Philips Healthcare, Best, The Netherlands) and 3) 3 T Toshiba Vantage Titan (Toshiba Medical Systems Corporation, Otawara, Japan, now part of Canon Medical). All exams were scanned by a trained post-doc (HA). The acquired images were 3D T1w (see Table 1) and only on the GE a single additional 3D Fluid Attenuation Inversion Recovery (FLAIR) was scanned, (TE/TR/TI = 130/8000/2340 ms and 1.0 × 1.0 × 1.2 mm^3^), using acquisition protocols optimized locally. The protocols were optimized for diagnostic purposes for the same group of radiologists for each scanner. The aim of this study is to investigate if brain volume is affected by scanner differences in a clinical setting. Therefore, we did not standardize each TE, TR and TI between scanners. In the current study we will use the term between-scanner to encompass both the differences arising from using different scanners and the variations resulting from different acquisition protocols. By using “between-scanner” in this manner, we aim to acknowledge and account for the combined influence of both factors on the observed volumes. For all acquisitions distortion correction was applied.

**Table 1:** MRI acquisition parameters: FOV = Field of View, TR = Repetition Time, TE = Echo Time, TI = Inversion Time, FSPGR = Fast SPoiled GRadient Echo, TFE = Turbo Field-Echo, FFE = Fast Field Echo

Scanner	Slices	FOV (mm^2^)	Pulse sequence name	Orientation	Resolution (mm^3^)	TR (ms)	TE (ms)	TI (ms)	FA (º)
GE Discovery MR750	172	256 × 256	FSPGR	sagittal	1.0 × 1.0 × 1.0	8.2	3.2	450	12
Philips Ingenuity	176	256 × 256	TFE	sagittal	1.0 × 1.0 × 1.0	7.9	4.5	900	8
Toshiba Vantage Titan	176	256 × 256	FFE	sagittal	1.0 × 1.0 × 1.2	5.7	2.4	1050	9

### Lesion filling

**Fig. 1:**
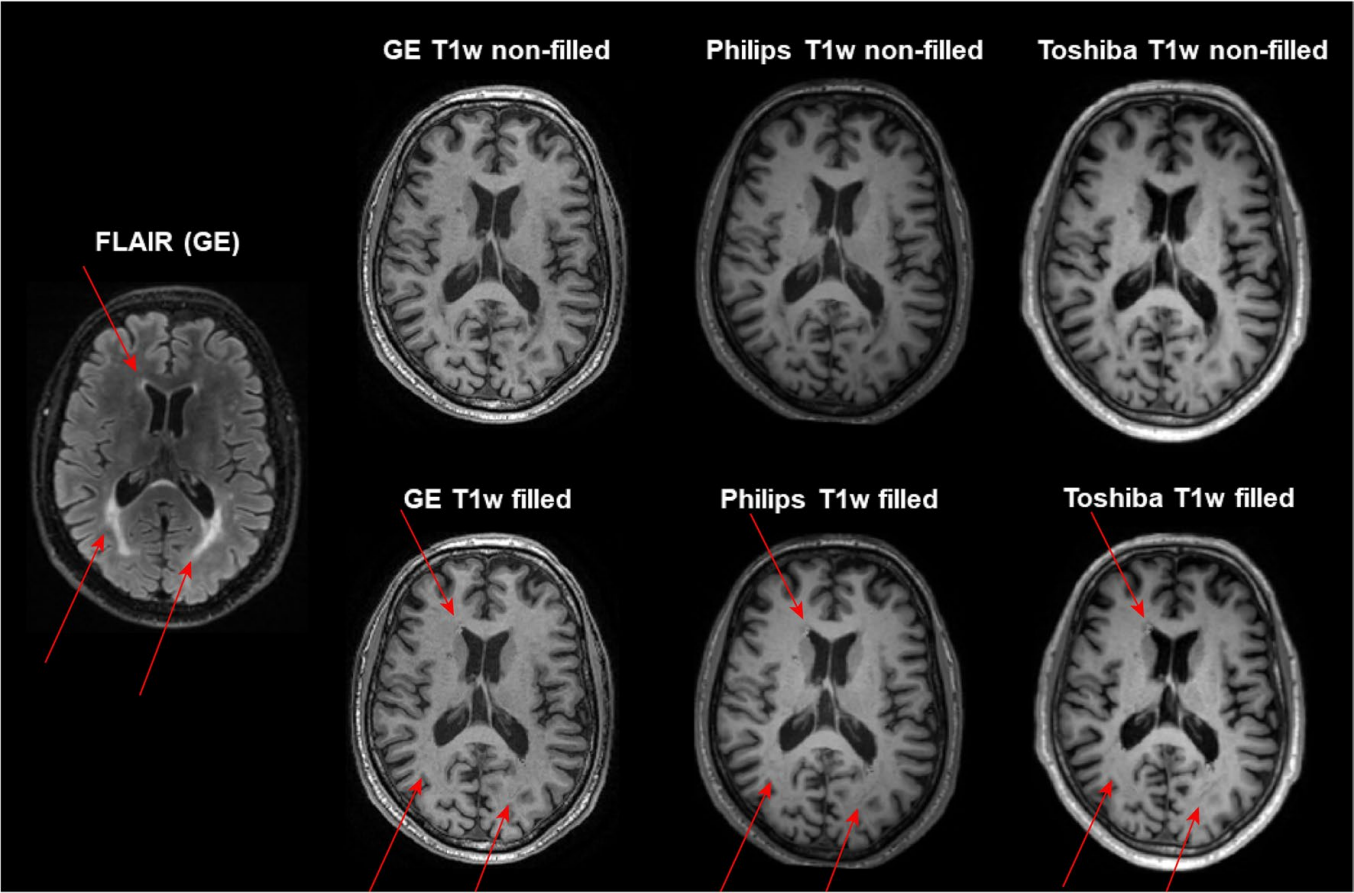
Example of non-filled and filled T1w images and the additional FLAIR scan of the same subject. Red arrows indicate the lesions on FLAIR and the filled lesions on the T1w images

To avoid potential variation due to MS lesions, lesions were filled on 3D T1w images [31]. Lesion segmentation was performed on the 3D FLAIR images using nicMS lesions (https://github.com/sergivalverde/nicMSlesions), which resulted in an individual lesion probability map for each patient in their 3D-FLAIR space. Afterwards, these were linearly registered to the six different 3D T1 spaces of the same patient using FMRIB's Linear Image Registration Tool (FLIRT) (Jenkinson & Smith, 2001), with 12 degrees of freedom, mutual information as the cost function and trilinear interpolation. Subsequently, a threshold of 0.5 was applied to obtain the lesion mask. Then, both the 3D T1w and lesion mask images were used to fill lesion on T1w images with the help of lesion automated preprocessing (LEAP) [32]. An example of the FLAIR and non-filled and filled T1w images is given in Fig. 1.

### Brain volumetry

**Fig. 2:**
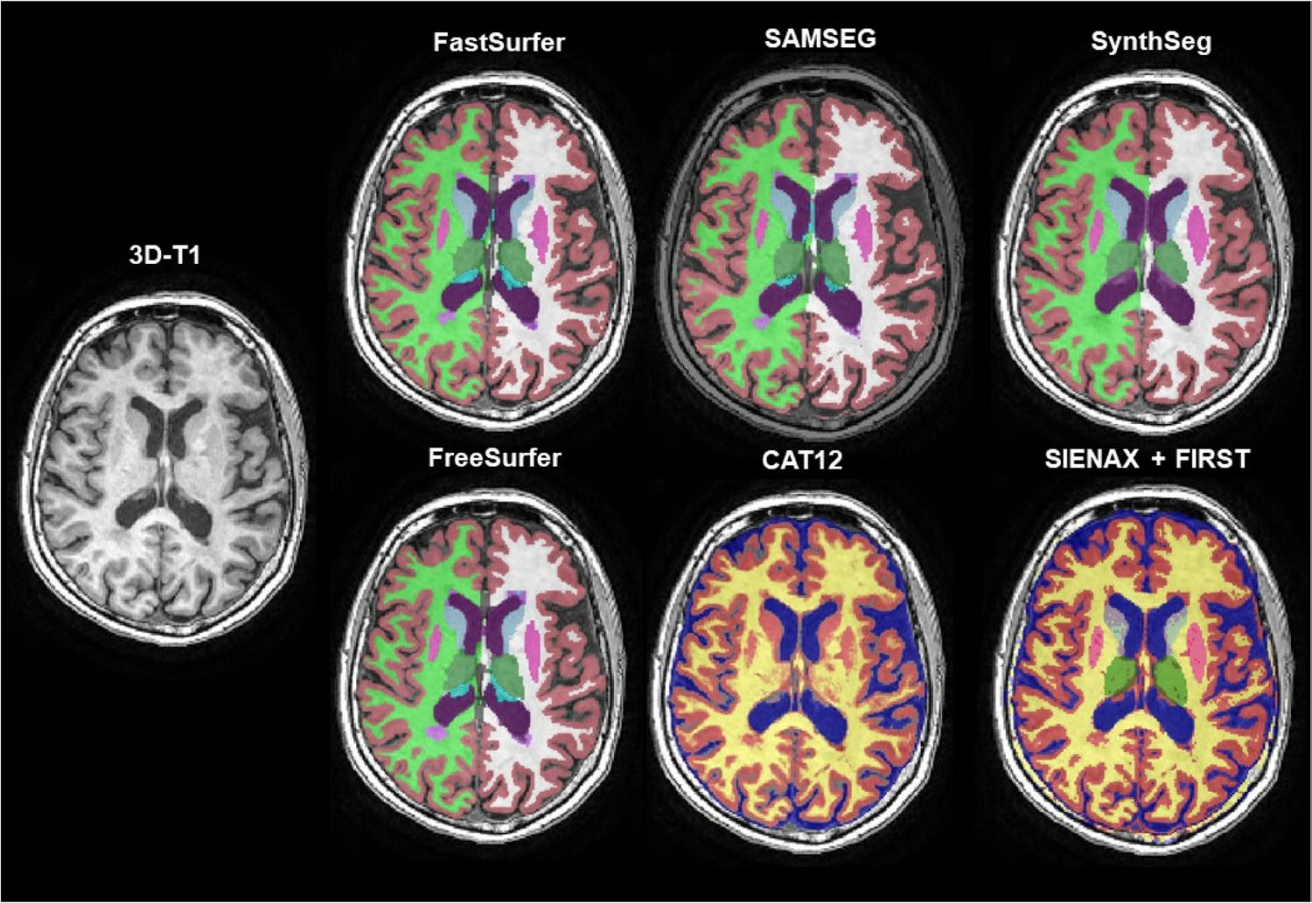
Example of images of the GE scanner and corresponding segmentations for one subject and the first scan (non-filled). Please note that CAT12 and SIENAX + FIRST have different color scales

Both non-filled and lesion-filled images were segmented into regions of interest (ROIs) with the segmentation methods described in Sect. 2.4.1 to 2.4.7. For this study, we looked at whole-brain, white matter (WM),^1^ gray matter (GM), cerebrospinal fluid (CSF) and bilateral deep grey matter (DGM) (amygdala, nucleus accumbens, caudate nucleus, hippocampus, pallidum, putamen and thalamus) volume. If necessary, additional preprocessing (such as neck removal) was performed. For additional analyses, we concentrated results to only whole brain, GM, WM, CSF and the thalamus volumes. Quality control (QC) of the segmentations was performed on a randomly selected set of images. An example of the segmentations is shown in Fig. 2.

#### CAT-12

The Computational Anatomy Toolbox (CAT) 12 version 1830 (http://www.neuro.uni-jena.de/cat/index.html, Jena University Hospital, Jena, Germany) was used, which is an addition to SPM-12 (http://www.fil.ion.ucl.ac.uk/spm/software/spm12/, Wellcome Trust Centre for Neuroimaging) running in Matlab R2018b (The MathWorks, Natick, MA) [33]. Briefly, CAT-12 uses a combination of a priori tissue probability maps for normal subjects and an intensity-based tissue classification to increase the accuracy of the segmentation of an MR image into GM, WM and CSF [34]. The neck and skull are stripped automatically before segmentation. The brain volume in CAT-12 was defined as the sum of the GM and WM volumes and the total intracranial volume (TIV) was used for the normalisation for head size*.* No additional pre-processing or manual intervention was performed. The cross-sectional data segmentation tool was run using the default settings including segmentation of the DGM structures using the Hammers atlas [35].

#### FreeSurfer

FreeSurfer version 7.1.1 was used; a detailed description can be found here: https://surfer.nmr.mgh.harvard.edu/fswiki/ and has previously been described [23, 36]. In short, both volume-based and surface-based approaches are used to produce volume measurements of the brain. It applies several automatic preprocessing steps such as skull stripping, intensity normalization and bias field correction. FreeSurfer presents the estimated TIV (eTIV), which is based on the relationship between the intracranial volume (ICV) and the linear transform to MNI305 space, as a normalization measure [37]. However, this relationship is biased by brain volume [38] and may therefore result in incorrect normalization. Therefore, we also normalized FreeSurfer volumes with the segmentation-based estimate of the TIV (sbTIV) derived from SAMSEG. FreeSurfer was run with the -3T -all options.

#### SAMSEG

Sequence Adaptive Multimodal SEGmentation (SAMSEG) (https://surfer.nmr.mgh.harvard.edu/fswiki/Samseg) is a relatively new approach that has been described in [29]. In brief, SAMSEG uses a probabilistic atlas which is mesh-based and does not require any preprocessing steps. The segmentation-based estimate of the TIV (sbTIV) was used for normalization. SAMSEG is part of the FreeSurfer package.

#### FSL

FSL-FIRST was used for DGM segmentation and FSL-SIENAX for WM, GM, CSF and whole brain segmentation both from FSL version 6.0.4 (https://fsl.fmrib.ox.ac.uk/fsl/fslwiki/) [24, 25, 39]. FSL-FIRST, a model-based tool, uses shape and appearance models which were derived from a large dataset. SIENAX separately estimates the GM, WM peripheral GM and ventricular CSF volume fractions [40]. Before this, a volumetric scaling factor is determined by registration of the skull image to the MNI 152 space [41]; this was used for normalization for both FIRST and SIENAX. As proposed by [42] the optimal parameters “-B -f 0.1”; and neck removal were applied for FSL-SIENAX in this study.

#### FastSurfer

FastSurfer (https://github.com/Deep-MI/FastSurfer) is a convolutional neural network (CNN) based on FreeSurfer output [26]. It was trained on 140 subjects which were processed with FreeSurfer version 6.0.0 and produces similar output. Additionally, the surface-based pipeline was run. No preprocessing was performed. The eTIV was used for normalization.

#### SynthSeg

Another CNN, called SynthSeg (https://github.com/BBillot/SynthSeg), tackles the generalizability problem of deep learning approaches by training on synthetic data which was sampled from manual and FreeSurfer segmentations and corresponding input images [28]. SynthSeg (v1.0) does not provide any normalization volume or estimate such as the eTIV or sbTIV, therefore sbTIV from SAMSEG was applied here.

##### Statistical analyses

**Fig. 3:**
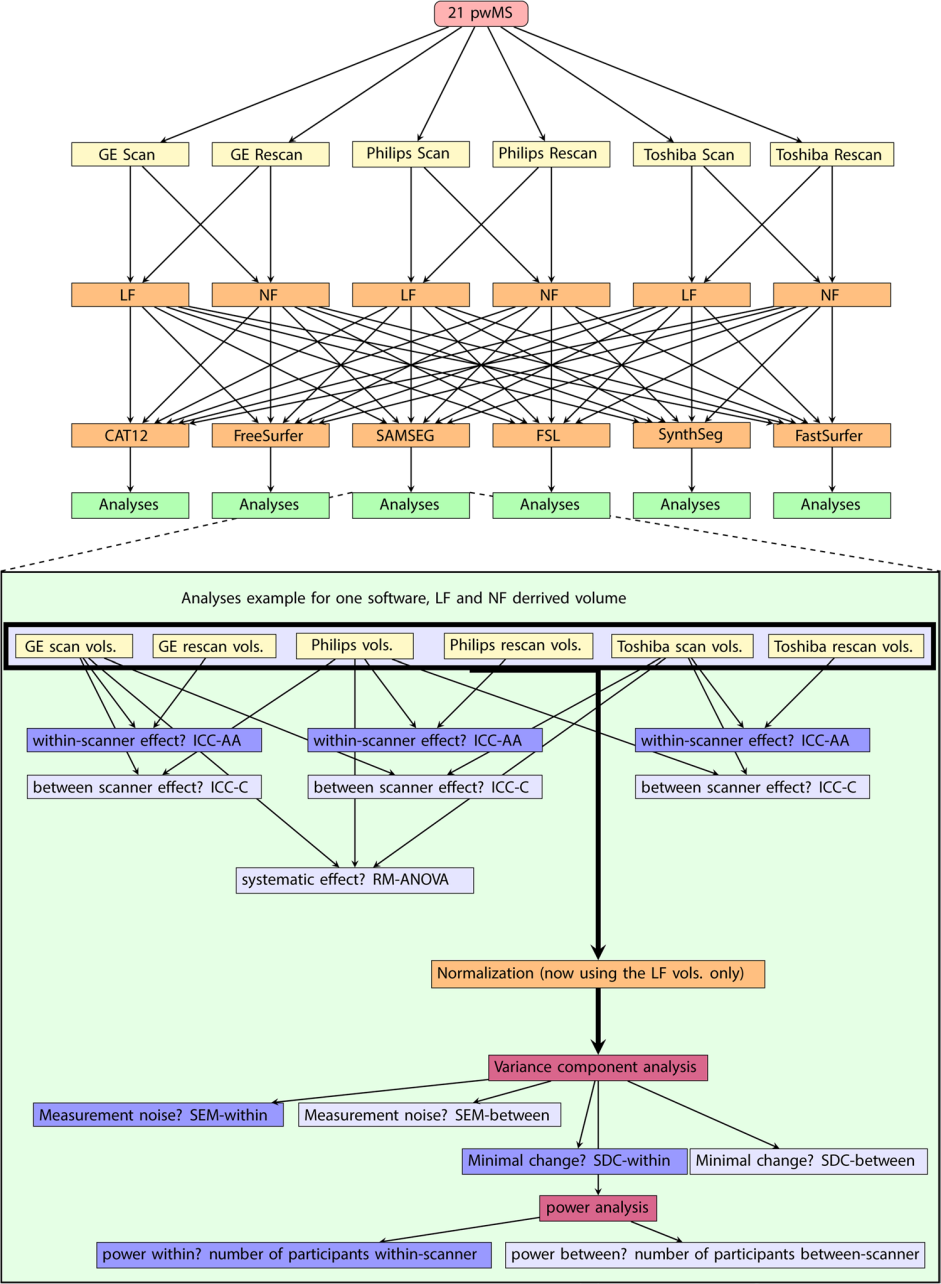
Schematic of measurements with accompanying tests. Yellow indicates input data, orange indicates a data processing step, light-blue indicates a repeatability (within-scanner) measure, dark-blue indicates a reproducibility (between-scanner) measure, purple indicates whole group analyses. pwMS = people with multiple sclerosis, LF = lesion-filled, NF = non-filled, vol(s) = volume(s) derived with the software, ICC = intra-class correlation coefficient, ICC-AA = ICC-absolute agreement, ICC-C = ICC-consistency, VCA = variance component analyses, SEM = standard error of measurement, SDC = smallest detectable change

All statistical analysis was performed using R Statistical Software (version 4.1.1; R Foundation for Statistical Computing, Vienna, Austria). An overview of all statistical analyses is depicted in Fig. 3. Both repeatability and reproducibility were assessed cross-sectionally with the intra-class correlation coefficient (ICC) with a 95% confidence interval (CI) for absolute agreement within scanner (ICC-AA) and for consistency between scanners (ICC-C), respectively. Note that the ICC-C does not reflect potential systematic difference between measurements. The ICC-values were classified according to the standards of Koo and Li (2016) [43]. ICC-C was tested on the first run of the scan-rescan images of all scanners. Reproducibility was assessed with a repeated measures ANOVA or a Friedmann test for not normally distributed data. If appropriate, post hoc testing was performed using pairwise t-tests or Wilcoxon signed rank tests. Reported *p*-values are Bonferroni corrected. The previous analyses were performed for un-normalized volumes to mitigate effects of improper normalization. For the following analyses we did normalize because this is common practice in a cross-sectional setting. With the normalized volumes a variance component analyses (VCA) was performed. From variance estimates we computed the standard error of measurement (SEM), as percentage of the mean, for within-scanner (SEM_within_) and between-scanner (SEM_between_) measurements [44]. Where SEM_within_ was defined as the square root of the residual variance (σ^2^_ε_) divided by the mean volume of the structure ($\overline{\mathrm{V} }$): ${\mathrm{SEM}}{\mathrm{within}}={~}^{\sqrt{{\upsigma }_{\upvarepsilon }^{2}}}\!\left/ \!{~}_{\stackrel{\mathrm{-}}{\mathrm{V}}}\right.\mathrm{100\%}$, and the SEM_between_ was defined as the square root of the sum of the rater (scanner) variance (σ^2^_r_) and σ^2^_ε_ divided by $\overline{\mathrm{V} }$: ${\mathrm{SEM}}{\mathrm{between}}\mathrm{=}{~}^{\sqrt{{{\upsigma }_{\mathrm{r}}^{2}}_{ }+{\upsigma }_{\upvarepsilon }^{2}}}\!\left/ \!{~}_{\stackrel{\mathrm{-}}{\mathrm{V}}}\right.\mathrm{100\%}$. Additionally we calculated the percentage smallest detectable change (SDC) from the SEM (SDC = 1.96 · $\sqrt{2}$ · SEM), which indicates the minimum percentage change considered to be a significant change (with 95% certainty) [44]. To assess any fixed and proportional bias, Bland-Altmann plots were created. These plots depict the volume difference between scanners as a function of the average volume with accompanying 95% CI. Moreover we executed a power analyses to evaluate the minimum group sizes needed to detect 1% difference in volume, using alpha = 0.05 and 80% power. These analyses were run for situations within-scanner or between-scanners.

## Results

### Demographics

The demographics of the MS patients are shown in Table 2. The second run of one subject on the GE scanner had clear motion artifacts so these images were excluded from the analyses.

**Table 2:** Demographics and clinical characteristics

	MS (*n* = 21)
Demographics at baseline	
Male, n (%)	6, (28%)
Age^a^, y (range)	47.7 ± 9.4 (32–60)
Disease duration^b^ (range)	11.38 ± 9.1 (1–40)
Disease-modifying treatment	
(none/TEC/COP/AVO/FIN/NAT/FAM), n	5/5/2/1/3/3/3/2

*Abbreviation: TEC* dimethyl fumarate (Tecifidera), *COP* glatirameer acetate (Copaxone), *AVO* interferon-β1 α (Avonex), *FIN* fingolimod, *NAT* natalizumab, *FAM* fampridine (Fampyra). ^a^ Mean ^b^ Mean since diagnosis.

### Reliability

**Fig. 4:**
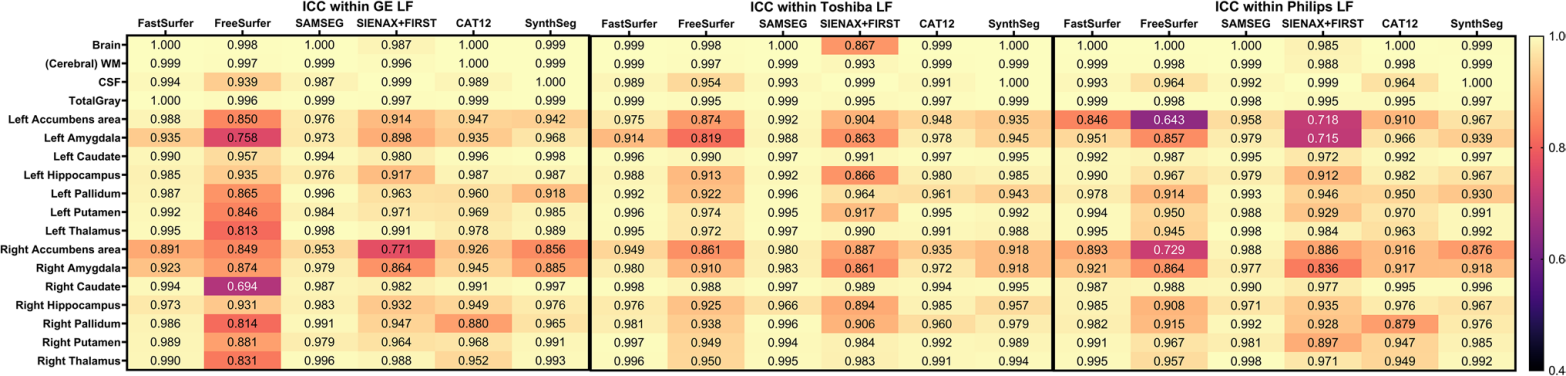
Heatmap of the within-scanner agreement (ICC-AA) for each scanner for the lesion-filled T1w images

**Fig. 5:**
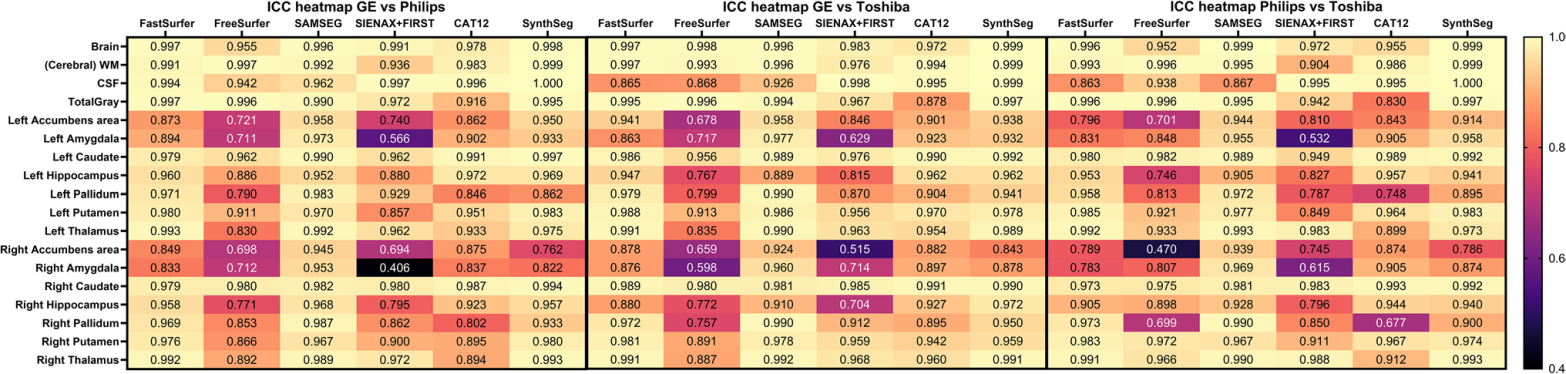
Heatmap of the between-scanner agreement (ICC-C) for all three pairwise scanner combinations for the lesion-filled T1w images

**Fig. 6:**
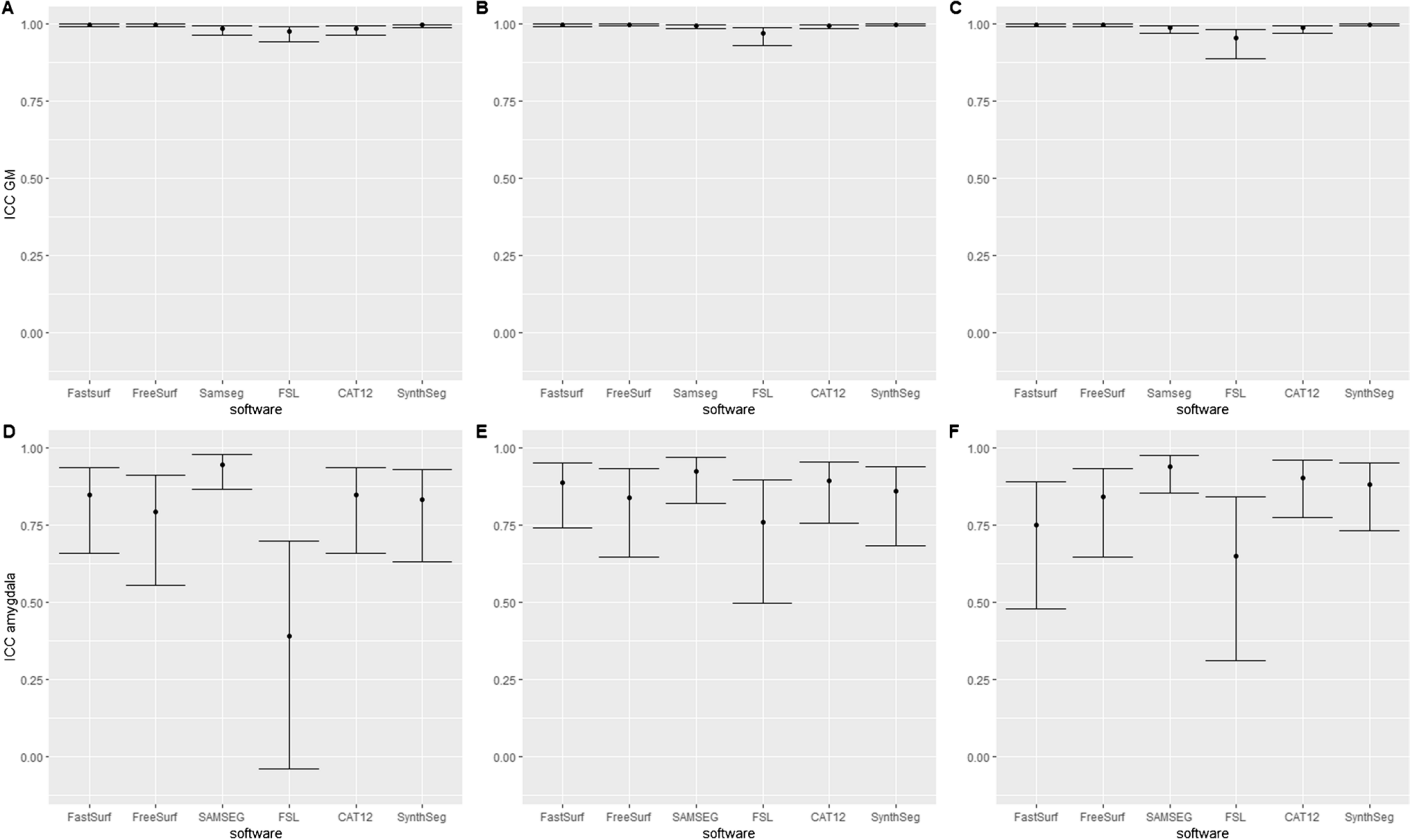
An example of the ICC-C values for lesion filled images with the corresponding 95% confidence interval for GM (A: GE vs Philips, B: GE vs Toshiba, C: Philips vs Toshiba) and the Amygdala (D: GE vs Philips, E: GE vs Toshiba, F: Philips vs Toshiba)

**Fig. 7:**
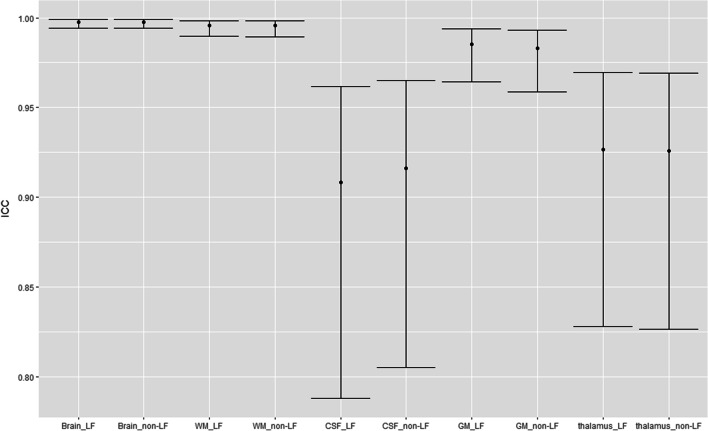
ICC-C scores for lesion filled (LF) and non-lesion filled (non-LF) with the 95% confidence interval for volumes of the Brain, CSF, GM, thalamus and WM segmented with CAT12 for GE vs Philips scanner

The within-scanner ICC-AA was above 0.9 for brain, GM, WM and CSF volumes segmented on both the lesion-filled as non-filled images, indicating excellent reliability (Fig. 4 and supplementary materials Fig. 1). However, for smaller (DGM) structures, which are typically more difficult to segment, the ICC-AA values ranged from 0.64 to 0.99 indicating moderate to excellent reliability. Generally, FreeSurfer and FSL have lower ICC-AA compared to other software. The overall between-scanner ICC-C was good to excellent (> 0.7), except for some small structures such as the accumbens (range: 0.4–1). Figure 5 shows that generally SAMSEG has the highest overall ICC-C followed by FastSurfer and SynthSeg. An example of the ICC for the amygdala with 95% CI is given in Fig. 6. The 95% CI of the amygdala ICC for FSL and SAMSEG do not overlap for this instance, but for the GM these intervals are very similar. For the same software and brain structure ICC-AA were higher than ICC-C between scanners (range: 0.65–1 vs. 0.41–1). The ICC values for the lesion filled and non-lesion filled images and their corresponding CI were highly overlapping (Fig. 7).

### Systematic differences between scanners

**Fig. 8:**
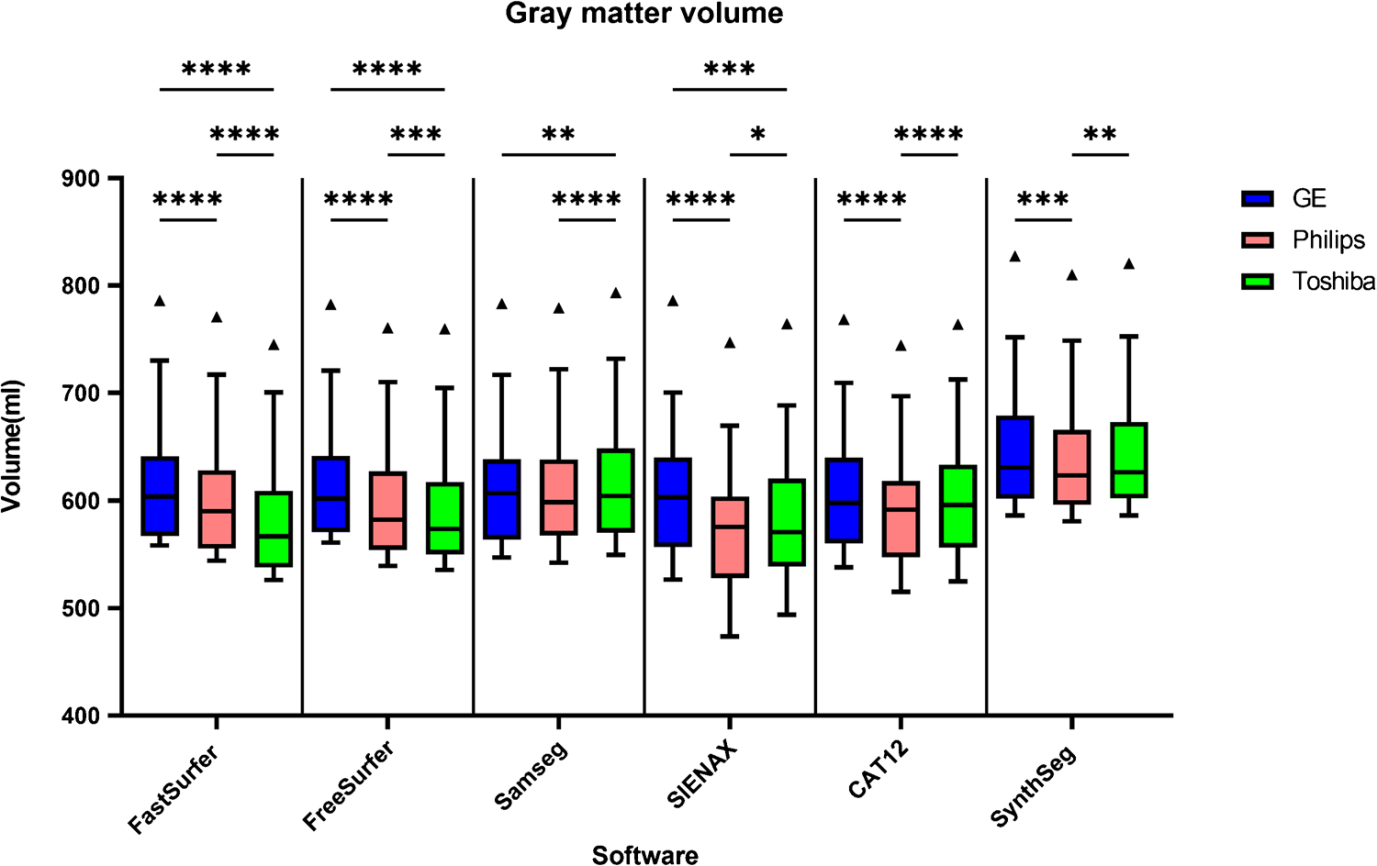
Boxplot (Tukey, line at median) of the total gray matter volume measurements grouped per scanner and software for lesion-filled images. * *p* < 0.05, ** *p* < 0.01, *** *p* < 0.001 **** *p* < 0.0001

**Fig. 9:**
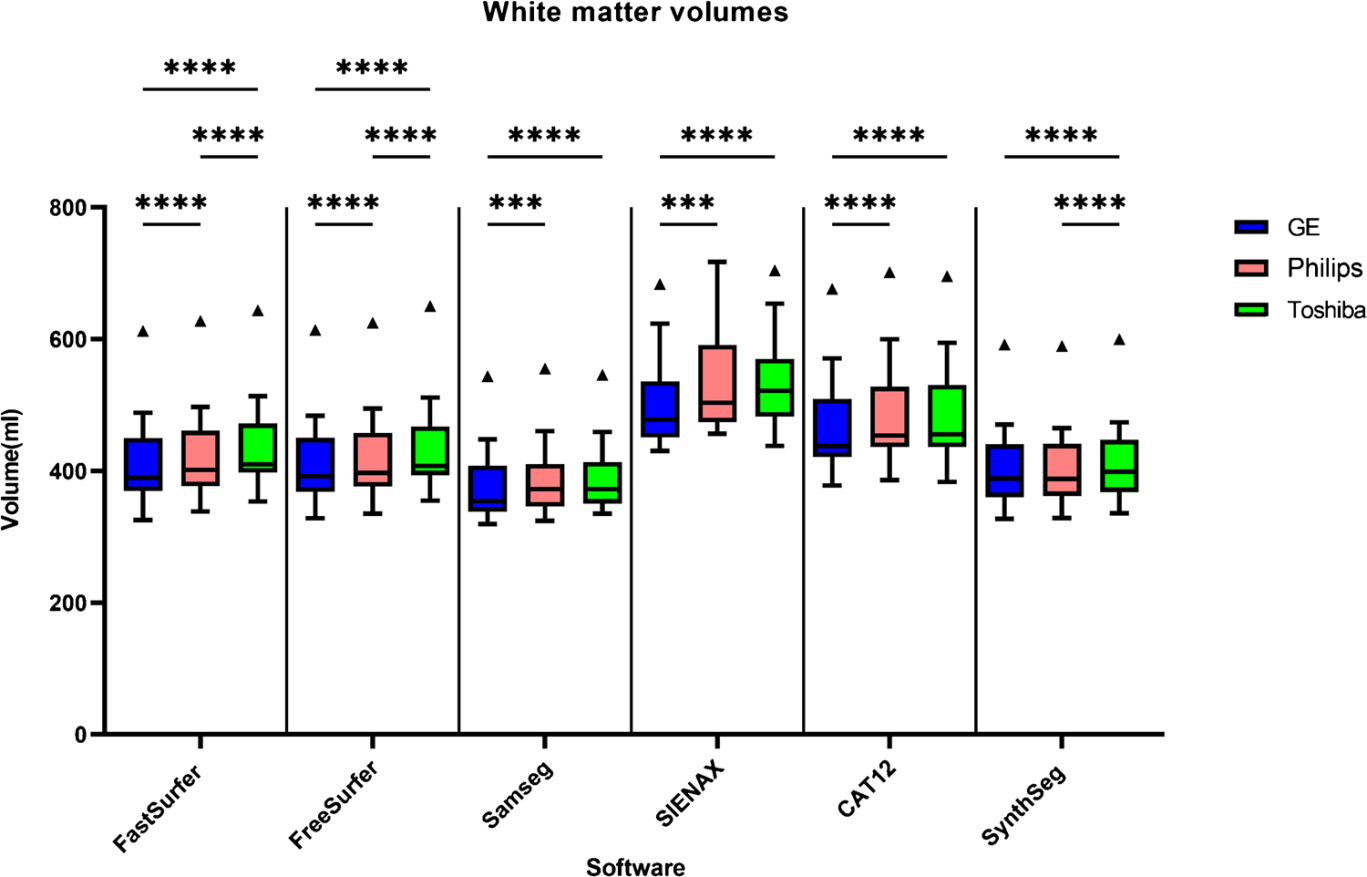
Boxplot (Tukey, line at median) of the white matter volume measurements grouped per scanner and software for the lesion-filled images. Note that the volumes for FastSurfer, FreeSurfer, SAMSEG and SynthSeg are lower because these only consider the cerebral white matter. * *p* < 0.05 ** *p* < 0.01 *** *p* < 0.001 ****p < 0.0001

Although the between-scanner reliability was high, systematic differences were found for all software packages in both GM and WM (Figs. 8 & 9). For every software package, the white matter for GE had a lower volume compared to Toshiba and Philips (*p* < 0.001, except for SynthSeg compared to Philips). Conversely, the volume of gray matter segmented from GE scans was higher compared to Philips (*p* < 0.001, except for SAMSEG). This was also true for GE vs. Toshiba for FastSurfer (*p* < 0.0001), FreeSurfer (*p* < 0.0001), SAMSEG (*p* < 0.01) and SIENAX (*p* < 0.001). Similar observations were found for the non-filled images (see supplementary materials). For whole-brain volume measurements differences were present depending on scanner and software except for FSL-SIENAX where there were no significant differences between scanners (supplementary materials Fig. 3).

### Variance component analyses

## Bland–Altman plots, standard error of measurement and smallest detectable change

In the Bland–Altman plots, although there was a fixed bias, no obvious proportional bias was observed. An example of the Bland–Altman plots is depicted in supplementary materials (Fig. 6) for GM, WM and CSF volumes segmented with SIENAX. Similar observations were found for other software. In Table 3, the SEM of the normalized volumes is detailed as percentage of the mean for each structure separately. As can be seen from the table, the SEM_between_ is between 1.75% and 12.42%, while the SEM_within_ is lower (range: 0.37–5.25%) for the same structure and software. Similar observations were found for the SDC for un-normalized volumes where, depending on the software and structure, the SDC for scans from between-scanner analyses was up to nine times higher compared to within-scanner analyses Table 4*.* In Tables 3 & 4, FreeSurfer volumes were normalized with the sbTIV because eTIV normalized volumes resulted in an increase of the SDC up to 19.28% (supplementary materials Table 1).

**Table 3:** Standard error of measurement as percentage of the mean. For a situation within-scanner (W) or between-scanners (B) (FreeSurfer was normalized by the sbTIV, since eTIV increased the SEM severely)

	FastSurfer		FreeSurfer		SAMSEG		FSL		CAT12		SynthSeg	
Comparison	W	B	W	B	W	B	W	B	W	B	W	B
Total brain	0.51	4.39	0.56	1.96	0.37	2.07	1.32	2.08	0.92	2.75	0.45	1.98
(Cerebral) WM	0.62	6.78	0.85	4.12	0.53	2.39	1.55	4.35	1.12	3.90	0.60	2.38
CSF	1.84	7.06	5.25	8.59	0.97	5.30	1.63	5.42	2.79	8.33	0.59	3.05
Total Gray	0.61	4.07	0.68	2.59	0.49	2.20	1.51	4.02	0.97	2.71	0.45	1.90
Thalamus	1.09	2.84	3.45	5.27	0.69	1.76	1.66	3.02	3.28	10.55	0.99	1.75

**Table 4:** Smallest detectable change as percentage of the mean. For a situation within (W) or between-scanner (B). (FreeSurfer was normalized by the sbTIV, since eTIV increased the SEM severely)

	FastSurfer		FreeSurfer		SAMSEG		FSL		CAT12		SynthSeg	
Comparison	W	B	W	B	W	B	W	B	W	B	W	B
Total brain	1.43	12.17	1.56	5.43	1.02	5.73	3.67	5.77	2.55	7.62	1.26	5.48
(Cerebral) WM	1.70	18.78	2.35	11.43	1.48	6.61	4.29	12.05	3.11	10.82	1.66	6.61
CSF	5.11	19.57	14.55	23.81	2.68	14.69	4.52	15.03	7.72	23.09	1.63	8.45
Total Gray	1.69	11.27	1.88	7.17	1.36	6.10	4.20	11.15	2.70	7.52	1.25	5.27
Thalamus	3.01	7.88	9.57	14.62	1.92	4.89	4.61	8.36	9.08	29.25	2.76	4.83

## Power analyses

**Fig. 10:**
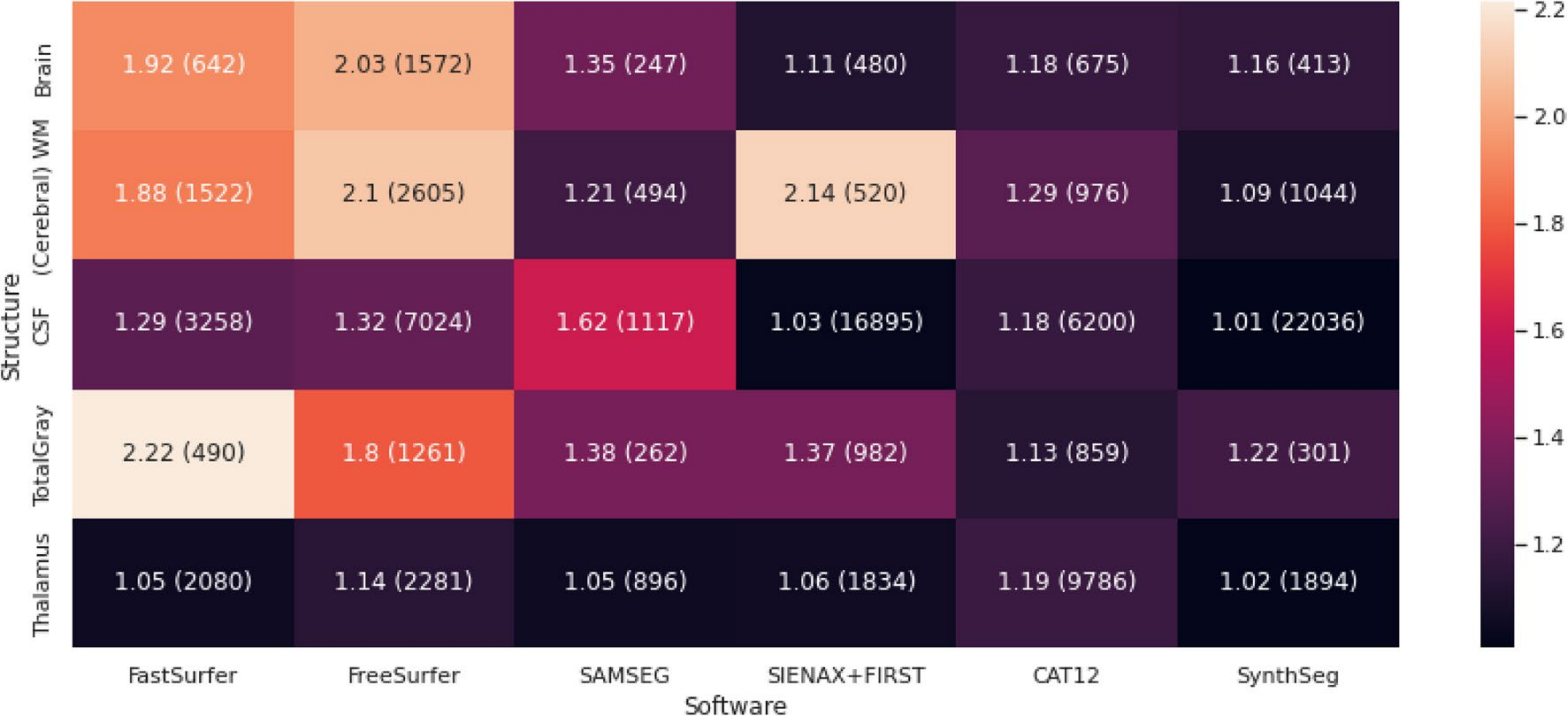
Heatmap of the ratio (between/within) of participants needed to detect 1% difference in (normalized) volume for a situation between (vendor) different MR scanners or within-scanner. In brackets the number of participants for a between-scanner situation is given. For example: for FastSurfer for between-scanner measurements 642 participants are needed to detect 1% difference in brain volume while for within-scanner measurements this would be: 334 (642/1.92 = 334)

A power analyses for sample size was conducted for between and within scanner measurements. In Fig. 10, the ratio of participants needed to detect 1% volume difference between groups for between scanner measurements or within scanner measurements is depicted. Depending on the ROI and on the software used, multiple scanner studies can yield a twofold increase in number of participants. Some structures are typically hard to segment so these result in an increase in participant number (e.g., the thalamus). For SynthSeg, the increase is less compared for example FreeSurfer or FastSurfer.

## Discussion

In this study, we provided a comparative study of 6 freely available software packages for brain volume segmentation in people with MS by examining within and between-vendor MR scanner effects. The main findings suggest that although the ICC scores were good to excellent, systematic differences between scanners were present between all the software packages examined. As expected, the within-scanner repeatability was higher than the between-scanner reproducibility. Lesion filling did not increase the ICC because of the highly overlapping 95% CI, but we did see erroneous segmentations in non-filled images, especially in and around lesions (supplementary materials Fig. 7). With the different error and correlation metrics used in this study, several different topics of interest were studied. A high ICC value indicates good reliability, meaning that subjects can reliability be distinguished from each other. This can occur either because the subjects are sufficiently different from each other, or because the influence of other sources of variation (also known as measurement error) is small enough [45]. In the presence of substantial between-subject variation, this measurement error may be large, but relatively small enough to still result in a high ICC. Therefore, the measurement error, expressed as the SEM, is informative, as it relates to the precision of the measurement. Furthermore, the RM-ANOVA and post hoc analyses provide insights into the presence of any systematic bias.

Given the relatively small atrophy rates (0.5–1.5% depending on structure en disease type) in people with MS, it is important that the measurement error is within the bounds of the volume loss to accurately obtain information on disease progression [7, 46]. We observed that for between-scanner measurements the SEM and SDC are significantly higher compared to within-scanner measurements. The estimates of the SEM (range: 0.37–1.32%) and SDC (range: 1.02–3.67%) suggest that the yearly total brain volume loss of a MS patient (0.5–1.35%) can be observed reliably on the same scanner within one or two years, while for between-scanner scanner situation it may take up to 5–10 years, depending on the structure of interest [46, 47]. Similar observations were found by Guo et. al [15] where they found that the coefficient of variation (CoV) was between 0.17–0.92% intra-scanner while the inter-scanner CoV was between 0.65 and 5.0%. In addition, Opfer et al. [48] also found that the within-scanner percentage difference was between 0.24% and 1.74% and found a tenfold increase for between scanner variability, which is in line with our results for the SEM. In contrast to the previous study, with the help of the SDC measure, information about the minimum change needed could be obtained. Our results concerning between-scanner differences are consistent with previous similar studies in MS [15, 49]. The lower ICCs, higher SEM and higher SDC for the smaller structures are likely due to the more difficult task of small ROI segmentation [50]. In addition, not segmenting, e.g., a WM-voxel on the GE scan while segmenting a voxel on the Philips scan has less influence on the ICC compared to the same situation for smaller structures.

The ICC analyses were performed on unnormalized volumes to exclude any effects of improper normalization on those results. However, for the calculation of the SEM and for the power analyses we did look at normalized volumes since normalization is usually applied in a cross-sectional study. Especially for the SEM, when the eTIV of FreeSurfer was used for normalization, the SEM increased up to 6.95% compared to normalization with the sbTIV. Volumes normalized with sbTIV resulted in similar SEM for FreeSurfer compared to other software. This suggests that the eTIV might not be a reproducible measure for normalization and other methods (e.g., sbTIV) have to be used when running FreeSurfer for segmentation, as suggested by FreeSurfer.

From all the segmentation methods, SAMSEG showed the lowest measurements error and highest between scanner reproducibility. Similarly, the amount of participants needed to detect a 1% difference in volume was less compared to other software. In addition, SynthSeg had similar performance compared to SAMSEG on the different metrics for reproducibility. Admittedly, most of the software had for example a lower SEM for GM (range: 0.49–1.51%) compared to the yearly GM atrophy rates (0.58–0.97%), assuming the measurement error does not increase over time. This was comparable for other structures [46]. However, the SDC increases systematically for between-scanners measurements. Both SAMSEG and SynthSeg have explicitly been developed to be adaptable to different scanners and moreover, different sequences [28, 29]. This suggests that for more reproducible results these types of software are preferable in contrast to the more traditional methods such as FreeSurfer and SIENAX. Noticeably, FastSurfer ICCs were higher than FreeSurfer, even though FastSurfer has been trained on FreeSurfer segmentation output instead of manual segmentations. It is worth mentioning that there are several more available software packages and that their accuracy assessment is warranted.

Even with the newest segmentation software, systematic differences between scanners persist. The goal of this research is not to provide one optimal software but to supply potential readers with a guideline to choose the optimal software depending on their input data and research purpose. Moreover, here we quantified the reproducibility in a multi-scanner setting for MS patients. Although the idea for data harmonization has been around for a long time and been adopted by initiatives such as the Alzheimer’s Disease neuroimage initiative this is generally not feasible in clinical practice [51]. Even with the harmonized approaches there is too much freedom for the protocol parameters, such as receiver coil, TE/TR/TI and k-space sampling strategy, impacting e.g. effective spatial resolution and CNR [52]. In addition, harmonization proposals to account for site and scanner effect such as the travelling brain approach, have their disadvantages [53]. A possible solution could be to provide phantoms with similar characteristics to human brains as proposed by [54].

### Limitations

Our study has some limitations. First, we had a relatively small sample size of 21 people with MS. Given this small sample size, we likely reported increased estimates of the variance compared to larger cohort studies. However, patients were scanned on 3 scanners and two scans on each scanner resulting in a total of 126 T1w scans for within patient comparisons. Secondly, we only looked at 3 T field strength. Currently, both 3 T and 1.5 T systems are frequently used in daily clinical care and tissues have different T1 and T2 relaxation times on 3 T versus 1.5 T systems which likely affects the segmentation [55, 56]. We unfortunately did not have manually outlined segmentations to check the quality of the segmentation of the images. In addition, not every segmentation was visually inspected for correctness. Third, because the FLAIR images were only acquired on the GE scanner, registration may have influenced the accuracy of the lesion filling pipeline. Moreover, potential between-scanner differences of FLAIR images could influence the T1w lesion-filling. However, among the many sources of variance (e.g., movement of the participant, scanner, protocol, software etc.) in this study we chose not to introduce an additional source of variance such as difference in the FLAIR protocol and differences in lesion segmentation. Fourth, this study was conducted in one center and images were acquired by one technician with optimized protocols for the neuroradiologists of the specific center.

## Conclusion

We demonstrated high within- and between scanner ICCs for brain volume measurements on T1w in MS, though systematic differences between scanners are present for every scanner and every software, depending on the structure. This implies that for a clinical setting or a cross-sectional multi-center/multi-scanner study, the effects of scanner need to be taken into account. Furthermore, to apply atrophy measurements in a clinical setting standardization of volume measurements in MS is needed.

## Supplementary Information

Below is the link to the electronic supplementary material.

Supplementary file1 (DOCX 997 KB)

## Data Availability

The data that support the findings of this study are available upon reasonable request from the author, [HV]. The data are not publicly available due to privacy restrictions of the research participants.

## Footnotes

1 For SAMSEG, FreeSurfer, SynthSeg and FastSurfer only cerebral WM was segmented.
