# Supplementary material for: Reliability of brain atrophy measurements in multiple sclerosis using MRI: an assessment of six freely available software packages for cross-sectional analyses
Source: Neuroradiology. 2023 Aug 1;65(10):1459–72. doi: 10.1007/s00234-023-03189-8 (PMC10497452; doi:10.1007/s00234-023-03189-8)
Supplement: Supplementary file 1 — Supplementary file1 (DOCX 997 KB) [file 234_2023_3189_MOESM1_ESM.docx]

# **Supplementary materials**


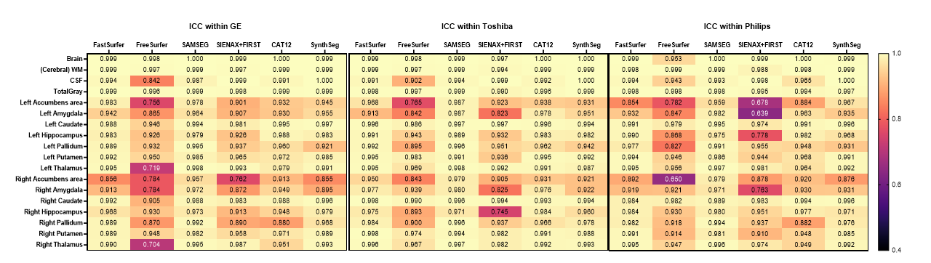


**Suppl. Fig. 1**. Heatmap of the within-scanner agreement (ICC-AA) for each scanner for the non-filled images.


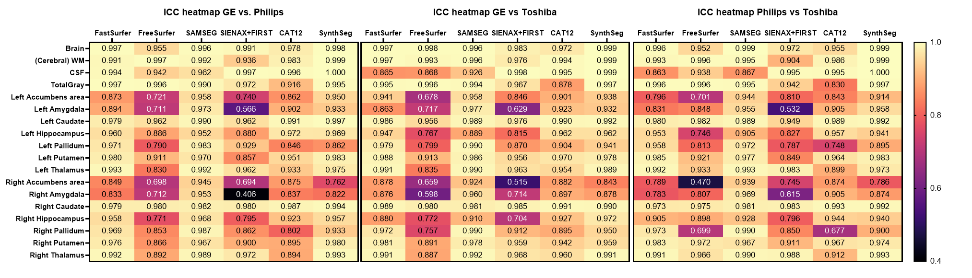


**Suppl. Fig. 12** Heatmap of the ICC for Consistency (95% confidence interval) for all three pairwise scanner combinations for the non-filled T1w images.


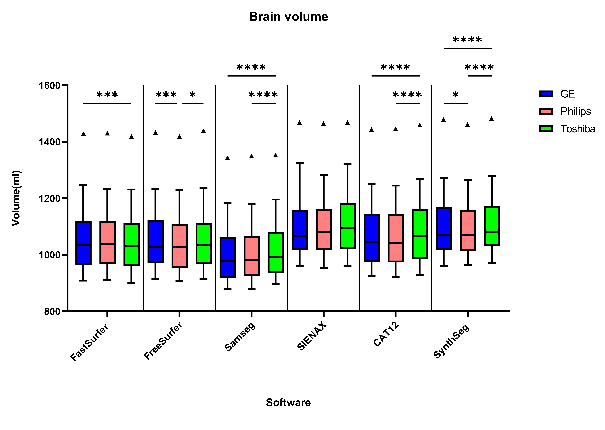


**Suppl. Fig. 13** Boxplot (Tukey, line at median) of whole-brain volume measurements grouped per scanner and software for the lesion-filled images. * p<.05, ** p<.01, *** p<.001.


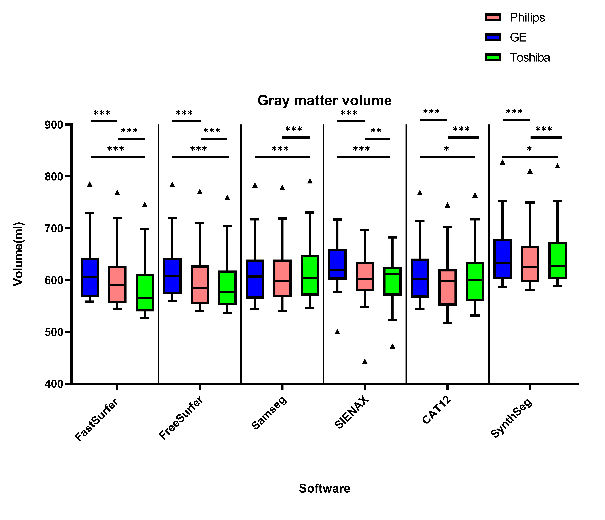


**Suppl. Fig. 14**. Boxplot (Tukey, line at median) of the total gray matter volume measurements grouped per scanner and software for the non-filled images. * p<.05, ** p<.01, *** p<.001.


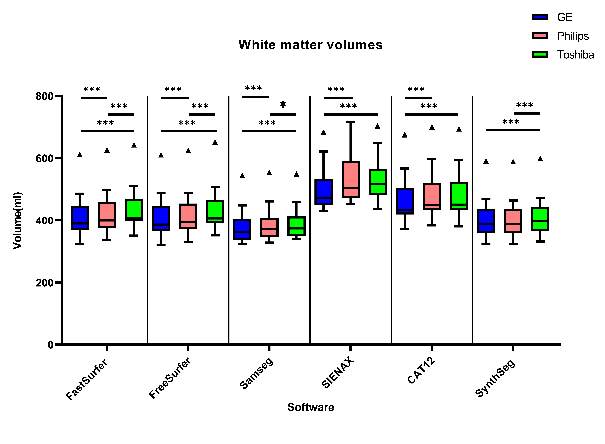


**Suppl. Fig.** **15.** Boxplot (Tukey, line at median) of the white matter volume measurements grouped per scanner and software for the non-filled images. Note that the volumes for FastSurfer, FreeSurfer, SAMSEG and SynthSeg are lower because these only consider the cerebral white matter. * p<.05 ** p<.01 *** p<.001.


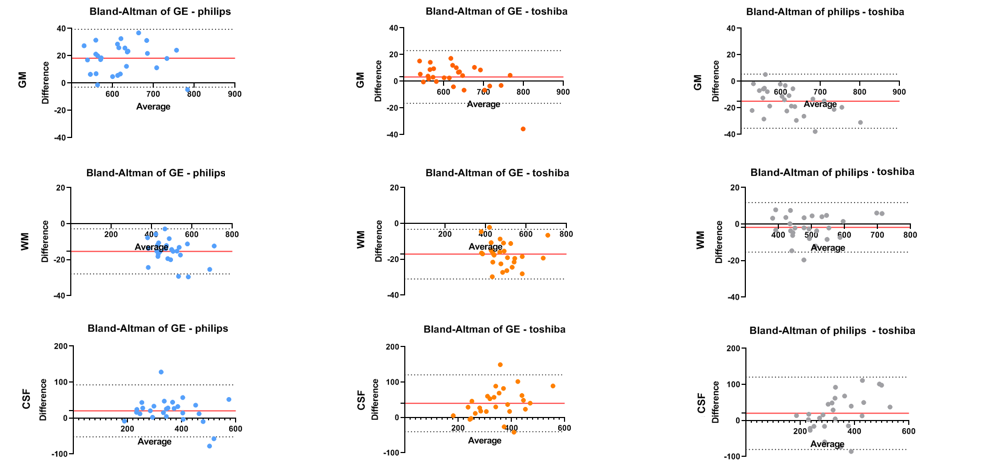


**Suppl. Fig. 16**. Bland-Altman plot showing the agreement between scanners for GM, WM and CSF volumes segmented with SIENAX. Depending on the structure a fixed bias can be observed. However, we did not observe a clear proportional bias.

**Table 5**. SEM and SDC for FreeSurfer volumes normalized with the eTIV and the sbTIV for within (W) and between (B) scanner measurements.

|  | FreeSurfer SEM_eTIV_ | | FreeSurfer SEM_sbTIV_ | | FreeSurfer SDC_eTIV_ | | FreeSurfer SDC_sbTIV_ | |  |
| --- | --- | --- | --- | --- | --- | --- | --- | --- | --- |
| Comparison | W | B | W | B | W | B | W | B | |
| Total brain | 5,60 | 8,91 | 0,56 | 1,96 | 15,52 | 24,71 | 1,56 | 5,43 | |
| (Cerebral) WM | 5,25 | 10,61 | 0,85 | 4,12 | 14,54 | 29,42 | 2,35 | 11,43 | |
| CSF | 6,90 | 12,44 | 5,25 | 8,59 | 19,13 | 34,48 | 14,55 | 23,81 | |
| Total Gray | 5,84 | 8,29 | 0,68 | 2,59 | 16,19 | 22,97 | 1,88 | 7,17 | |
| Thalamus | 6,68 | 8,33 | 3,45 | 5,27 | 18,52 | 23,10 | 9,57 | 14,62 | |

sbTIV = segmentation based total intracranial volume; eTIV estimated total intracranial volume; SEM = standard error of measurement SDC = smallest detectable change.


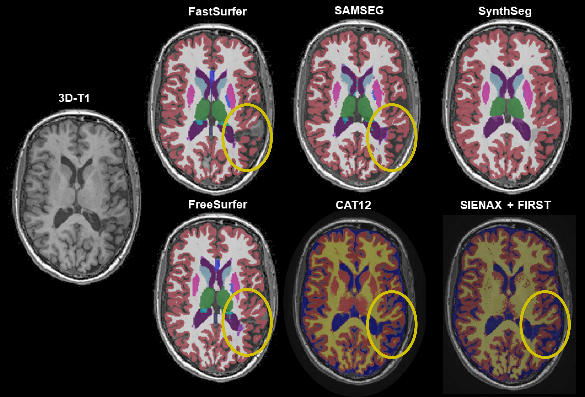


**Suppl. Fig. 17**. Example of segmentations for 1 subject on the 3T GE scanner and non-filled first scan. Yellow circles indicate that segmentation indicated different classification than expected, or because of presence of lesions.
